# Label-Free Biosensor

**DOI:** 10.3390/bios13050556

**Published:** 2023-05-18

**Authors:** Pengfei Zhang, Rui Wang

**Affiliations:** 1 Beijing National Laboratory for Molecular Sciences, CAS Key Laboratory of Analytical Chemistry for Living Biosystems, Institute of Chemistry, Chinese Academy of Sciences, Beijing 100190, China; 2 State Key Laboratory of Digital Medical Engineering, School of Biological Science & Medical Engineering, Southeast University, Nanjing 210096, China; ruiwang938@seu.edu.cn

Label-free biosensors have become an indispensable tool for analyzing intrinsic molecular properties, such as mass, and quantifying molecular interactions without interference from labels, which is critical for the screening of drugs, detecting disease biomarkers, and understanding biological processes at the molecular level. In the last decade, novel label-free imaging biosensors, including surface plasmon resonance microscopy, interferometric scattering microscopy, plasmonic scattering microscopy, and evanescent scattering microscopy, have further advanced this field by pushing beyond ensemble averages to reveal the statistical distributions of molecular properties and binding processes at the single-molecule level. These imaging techniques pave the way to understanding molecular interaction processes in great detail. Nonetheless, the demand for developing novel single-molecule, label-free sensing schemes that are cost-effective, easy to use, and especially applicable in commercial microscopy or other commercial label-free biosensors is ever increasing. Moreover, the application range of label-free biosensors is highly likely to expand, thus providing more powerful economical tools for clinical diagnosis, environmental monitoring, and industrial quality control. Therefore, this Special Issue, entitled “Label-free Biosensors”, focuses on recent advances in producing sensitive and easy-to-use label-free biosensors and their applications in diverse fields.

There are 11 peer-reviewed papers collected in this Special Issue. Six articles are included to show the development and applications of novel label-free biosensors. Xu et al. [1] developed a sequence-specific visualization method based on loop-mediated isothermal amplification for the detection of Salmonella—one of four key global causes of diarrhea—in milk. This method does not involve any sophisticated instrument; thus, it may be a useful tool in resource-limited areas. Mayer et al. [2] developed a sandwich-type electrochemical immunosensor for the quantitative detection of the carcinoembryonic antigen, an important tumor marker in clinical tests, using the redox-tagged, single-walled carbon nanohorns/thionine/AuNPs. The results showed that carbon nanohorns possess great potential for clinical diagnostics of CEA and other biomarkers. Cui et al. [3] developed a strong optical second-harmonic generation of a monolayer 2D semiconductor to observe interfacial molecular adsorption and desorption dynamics in a label-free manner. The proposed detection scheme principally undertakes a nanometer-scale spatial resolution across interfaces, which provides a powerful tool for understanding the spatiotemporal transports of matter and energy across interfaces. Nayak et al. [4] developed a label-free immunosensor by synthesizing carbon dots (CDs) with a one-step hydrothermal method and then covalently functionalizing the dots with antibodies for the sensing of progesterone hormone. This label-free immunosensor has emerged as a potential platform for simplified progesterone analysis due to the high selectivity performance and good recovery in different samples of spiked water. Zhao et al. [5] developed an aptamer-based fluorescence anisotropy sensor for rapid and sensitive detection of heavy metal cadmium ions, which is of great significance to food safety and environmental monitoring. This aptamer-based sensor works in a direct format for detection without the need for labeling in real water samples, showing broad application potential. He et al. [6] developed a smartphone-based biosensor for detecting human total hemoglobin concentration in vivo with high accuracy. The smartphone-based sensor utilizes the camera, memory, and computing power of the phone, thus largely reducing the cost. The authors demonstrated that this sensor could meet the accuracy requirements for non-invasive detection of hemoglobin concentration. Five reviews are also included to introduce recent advances in typical label-free biosensor families. Lei et al. [7] reviewed the development and applications of field-effect transistor (FET)-based biosensors, which have shown great technical potential in the biomarker detection platform. This mini review gives an overview of the design strategies of biosensors and then focuses on several representative aspects. Finally, the authors summarize the long-term prospects for the commercialization of FET sensing systems. Kuralay et al. [8] reviewed the label-free cancer diagnosis platforms using electrochemical methods for cancer diagnosis. The classification of the sensing platforms is generally presented according to their recognition element, and the most recent achievements of these attractive sensing substrates are described in detail. Cheung et al. [9] reviewed the biofunctionalization needs and strategies—which are inextricably linked to sensor performance—for silicon photonic (SiP) biosensors, a promising platform for robust and low-cost decentralized diagnostics. The authors evaluated the adsorption, bioaffinity, and covalent chemistries for immobilizing bioreceptors. Then, different biopatterning techniques were compared for spatially controlling and multiplexing the biofunctionalization of SiP sensors. Fang et al. [10] reviewed optical methods such as surface-enhanced Raman scattering spectroscopy, surface plasmon resonance, and dark-field microscopic imaging techniques for the rapid detection of pathogenic bacteria in a label-free manner. The advantages and disadvantages of these label-free technologies for bacterial detection are summarized in order to promote their application for rapid bacterial detection in source-limited environments and for drug resistance assessments. Feng et al. [11] reviewed the application of MXenes in electrochemical, optical, and other bioanalytical methods in recent years. The authors summarize and discuss problems in the field of biosensing and possible future directions of MXenes.

Label-free biosensors have been experiencing rapid development in recent decades and are becoming important tools for clinical diagnosis and environmental monitoring. A comprehensive review is thus necessary, and this is the main purpose of this Special Issues. Besides the review, this collection also presents several novel label-free detection techniques to demonstrate design strategies and operation protocols. Therefore, this Special Issue might be of particular value to beginners and graduate students who have just entered this field. We hope that they, through reading this collection of articles and reviews, might fully understand the principles and operation skills of label-free biosensors, and will go on to explore further the chemical and physical properties of the biosensors and their applications.

This Special Issue is the distillation of the authors’ intelligence and efforts. We expect that this collection will help and inspire researchers who are working in the fields of chemistry and biological science, and we also hope that it will provide a reference source or serve as a textbook for undergraduate and graduate students who major in chemistry, chemical engineering, physics, materials, and biology, as well as those readers who are otherwise interested in label-free biosensors. As this collection covers a breadth of highly diverse content connected to multiple scientific issues of some complexity, errors and omissions may not be entirely avoided; thus, we sincerely appreciate criticism and comments from readers.

## References

[1] Zeng H., Huang S., Chen Y., Chen M., He K., Fu C., Wang Q., Zhang F., Wang L., Xu X. (2023). Label-Free Sequence-Specific Visualization of LAMP Amplified Salmonella via DNA Machine Produces G-Quadruplex DNAzyme. Biosensors.

[2] Domínguez-Aragón A., Zaragoza-Contreras E.A., Figueroa-Miranda G., Offenhäusser A., Mayer D. (2023). Electrochemical Immunosensor Using Electroactive Carbon Nanohorns for Signal Amplification for the Rapid Detection of Carcinoembryonic Antigen. Biosensors.

[3] Xia C., Sun J., Wang Q., Chen J., Wang T., Xu W., Zhang H., Li Y., Chang J., Shi Z. (2022). Label-Free Sensing of Biomolecular Adsorption and Desorption Dynamics by Interfacial Second Harmonic Generation. Biosensors.

[4] Disha, Kumari P., Patel M.K., Kumar P., Nayak M.K. (2022). Carbon Dots Conjugated Antibody as an Effective FRET-Based Biosensor for Progesterone Hormone Screening. Biosensors.

[5] Yu H., Zhao Q. (2022). A Sensitive Aptamer Fluorescence Anisotropy Sensor for Cd2+ Using Affinity-Enhanced Aptamers with Phosphorothioate Modification. Biosensors.

[6] Fan Z., Zhou Y., Zhai H., Wang Q., He H. (2022). A Smartphone-Based Biosensor for Non-Invasive Monitoring of Total Hemoglobin Concentration in Humans with High Accuracy. Biosensors.

[7] Hao R., Liu L., Yuan J., Wu L., Lei S. (2023). Recent Advances in Field Effect Transistor Biosensors: Designing Strategies and Applications for Sensitive Assay. Biosensors.

[8] Sanko V., Kuralay F. (2023). Label-Free Electrochemical Biosensor Platforms for Cancer Diagnosis: Recent Achievements and Challenges. Biosensors.

[9] Puumala L.S., Grist S.M., Morales J.M., Bickford J.R., Chrostowski L., Shekhar S., Cheung K.C. (2023). Biofunctionalization of Multiplexed Silicon Photonic Biosensors. Biosensors.

[10] Wang P., Sun H., Yang W., Fang Y. (2022). Optical Methods for Label-Free Detection of Bacteria. Biosensors.

[11] Lu D., Zhao H., Zhang X., Chen Y., Feng L. (2022). New Horizons for MXenes in Biosensing Applications. Biosensors.

